# Biophysical physiology of phosphoinositide rapid dynamics and regulation in living cells

**DOI:** 10.1085/jgp.202113074

**Published:** 2022-05-18

**Authors:** Jill B. Jensen, Bjoern H. Falkenburger, Eamonn J. Dickson, Lizbeth de la Cruz, Gucan Dai, Jongyun Myeong, Seung-Ryoung Jung, Martin Kruse, Oscar Vivas, Byung-Chang Suh, Bertil Hille

**Affiliations:** 1 Department of Physiology and Biophysics, University of Washington, Seattle, WA; 2 Department of Neurology, University Medical Centre Carl Gustav Carus, Dresden, Germany; 3 Department of Physiology and Membrane Biology, University of California, Davis, Davis, CA; 4 Department of Biochemistry and Molecular Biology, Saint Louis University School of Medicine, St. Louis, MO; 5 Department of Cell Biology and Physiology, Washington University in St. Louis, St. Louis, MO; 6 Department of Chemistry, University of Washington, Seattle, WA; 7 Department of Biology and Program in Neuroscience, Bates College, Lewiston, ME; 8 Department of Brain Sciences, Daegu Gyeongbuk Institute of Science and Technology, Daegu, Republic of Korea

## Abstract

Phosphoinositide membrane lipids are ubiquitous low-abundance signaling molecules. They direct many physiological processes that involve ion channels, membrane identification, fusion of membrane vesicles, and vesicular endocytosis. Pools of these lipids are continually broken down and refilled in living cells, and the rates of some of these reactions are strongly accelerated by physiological stimuli. Recent biophysical experiments described here measure and model the kinetics and regulation of these lipid signals in intact cells. Rapid on-line monitoring of phosphoinositide metabolism is made possible by optical tools and electrophysiology. The experiments reviewed here reveal that as for other cellular second messengers, the dynamic turnover and lifetimes of membrane phosphoinositides are measured in seconds, controlling and timing rapid physiological responses, and the signaling is under strong metabolic regulation. The underlying mechanisms of this metabolic regulation remain questions for the future.

## Teaser

Phosphoinositides, rare membrane lipids, mediate powerful second messenger signals to ion channels, endocytosis, and exocytosis, and specify organelle identity. Jensen et al. summarize 20 yr of biophysical research whose key message is that pools of phosphoinositides turn over remarkably quickly to regulate rapid physiological responses.

## Introduction

This essay concerns a powerful membrane–lipid signaling system. We describe the kinetics, regulation, and physiological significance of cellular phosphoinositide lipid metabolism with special emphasis on the plasma membrane lipid, phosphatidylinositol 4,5-bisphosphate (PtdIns[4,5]P_2_; Balla, 2013; Mandal, 2020). Although they are low-abundance phospholipids, the phosphoinositides seem to have been selected by the stem eukaryotes as markers to distinguish the newly evolved variety of membrane compartments. PtdIns(4,5)P_2_ defines the eukaryotic plasma membrane like a postal ZIP code, and many plasma membrane proteins and processes read this code and require it to be active. Examples of central interest to physiologists include vesicular exocytosis, endocytosis, and the operation of numerous ion channels and transporters—many of which respond immediately to changes in membrane PtdIns(4,5)P_2_. Thus, modulation of PtdIns(4,5)P_2_ is a physiological signal that regulates key cellular processes. PtdIns(4,5)P_2_ satisfies all the criteria of a second messenger, although an unusual one since it is confined to the plasma membrane and, like cyclic guanosine monophosphate (cGMP) in retinal photoreceptors, it typically signals by concentration decreases. We undertook kinetic experiments with phosphoinositides because the dynamic changes of PtdIns(4,5)P_2_ concentration govern dynamic time courses of physiological responses, and an appreciation for rates and developing self-consistent kinetic descriptions are natural approaches for biophysicists.

This telling is personal and simplified. It marks the closing of our laboratory and focuses on questions we worked on but does not give full consideration to past literature or touch on many additional interesting topics of current phosphoinositide research. Some of the questions are only partly answered. One overarching conclusion is that the cellular pools of membrane phosphoinositides are highly dynamic. They turn over and change remarkably rapidly on a time scale of seconds to a few minutes. One might say that the fast turnover reflects the signaling role of phosphoinositides as second messengers and as location markers in a cellular environment of continual membrane remodeling.

## PtdIns(4,5)P_2_ and the phosphoinositide family

**Figure 1.**
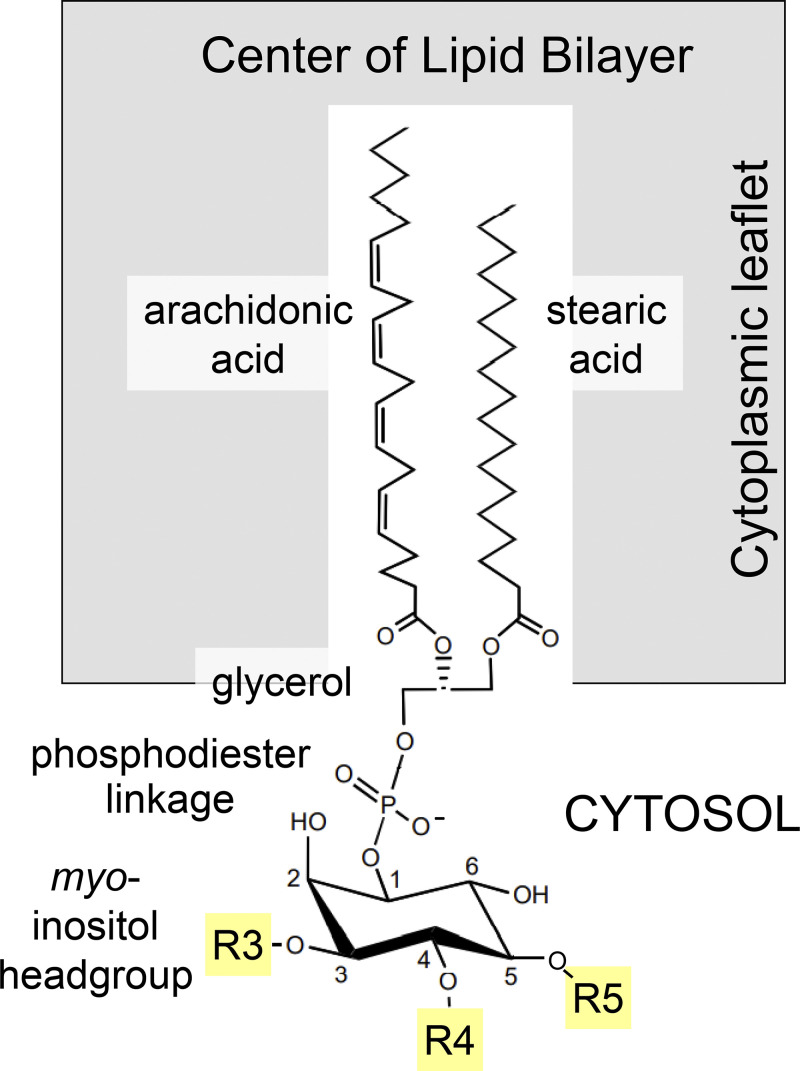
**Structure of mammalian phosphoinositides.** The three yellow positions (R3, R4, and R5) on the *myo*-inositol ring can be phosphorylated by lipid kinase enzymes and dephosphorylated by lipid phosphatases to yield seven phosphorylated combinations and permutations.

Like other typical membrane phospholipids such as phosphatidylcholine, phosphatidylserine, and phosphatidylethanolamine, the phosphoinositides contain a hydrophobic diacylglycerol moiety coupled by a phosphodiester linkage to a polar head group (Fig. 1). In this case, as for all eukaryotic phosphoinositides, the head group is *myo*-inositol, an isomer of the cyclic polyalcohol, hexahydroxy cyclohexane (Balla, 2013). Although, in all mammalian phosphoinositides, the most common fatty acid chains are arachidonic acid in the glycerol sn-2 position and stearic acid in the sn-1 position (Wenk et al., 2003; Hicks et al., 2006), other fatty acids are more prominent in e.g., flies, yeast, or slime molds (Randall et al., 2015; Wenk et al., 2003; Clark et al., 2014). The individual members of the phosphoinositide lipid family are distinguished by whether their inositol ring is phosphorylated on its 3-, 4-, and/or 5-position. All combinations and permutations are possible. They are interconverted by lipid kinases and lipid phosphatases acting on the exposed 3-, 4-, and 5-hydroxyls of the inositol ring. Each of these enzymes exists in multiple isoforms. They are mostly cytoplasmic but directed to specific target membranes by protein–protein interactions, and their substrate phosphoinositides reside in the cytoplasmic leaflets of organelles and the plasma membrane. PtdIns(4,5)P_2_, which is phosphorylated on the 4- and 5-hydroxyl positions (Fig. 1), resides almost exclusively in the cytoplasmic leaflet of the plasma membrane.

## Phosphoinositide metabolism

**Figure 2.**
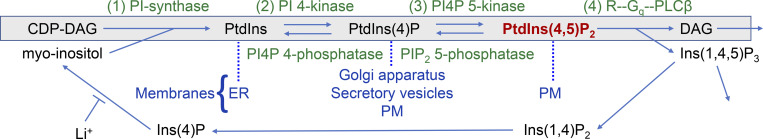
**Synthesis and breakdown of PtdIns(4,5)P**_**2**_ **(red).** This reaction diagram is often called the PI cycle. The lipid species on the gray membrane bar, PtdIns, PtdIns(4)P, etc., are found in the cellular membranes named below in blue. Enzymes are green. Almost all the enzymes have multiple isoforms differing in expression and localization. DAG, diacylglycerol; PM, plasma membrane; R–G_q_–PLCβ, receptor-activated PLCβ.

The enzymology and genetics of phosphoinositide metabolism are very well studied and reviewed including, for example, by the laboratories of Tamas Balla, Pietro de Camilli, and Scott Emr (Balla, 2013; Di Paolo and De Camilli, 2006; Odorizzi et al., 2000). One key finding is that the mix of phosphoinositides in each type of membrane in the cell is unique, the ZIP code idea. Phosphoinositide synthesis begins in intracellular membranes, mainly the ER, by coupling of cytoplasmic *myo*-inositol to an activated form of diacylglycerol (CDP-diacylglycerol) in the membrane, yielding phosphatidylinositol (PtdIns; Fig. 2, step 1). Relative to the total cellular phosphoinositide pools, PtdIns is by far the most abundant and constant. It is the precursor of all polyphosphorylated phosphoinositides. Addition of a 4-phosphate by PI 4-kinase enzymes generates phosphatidylinositol 4-phosphate (PtdIns(4)P) in the Golgi apparatus, secretory vesicles, and plasma membrane, a reaction requiring ATP (step 2). Further addition of a 5-phosphate by PI4P 5-kinase enzymes generates PtdIns(4,5)P_2_ in the plasma membrane (step 3). Corresponding lipid 4- and 5-phosphatases reverse these phosphorylations. In addition, and important here, PtdIns(4,5)P_2_, and possibly PtdIns(4)P, can be broken down by the receptor-activated enzyme phospholipase Cβ (PLCβ; Fig. 2, step 4). The products are hydrophobic diacylglycerol in the plasma membrane and the highly hydrophilic former head group inositol trisphosphate (Ins[1,4,5]P_3_) in the cytoplasm. We focus here on the phospholipase that is activated by G-protein coupled receptors (PLCβ), but in many circumstances, the same reaction is activated by receptor tyrosine kinases (PLCγ) or by elevated intracellular free calcium (PLCδ). A balance of the phosphoinositide pools in Fig. 2 and their enzymatic interconversions is so important for cellular functions that mutations of any of the enzymes have severe developmental consequences and may lead to pathological conditions including major anatomic malformations, areflexia, midbrain–hindbrain malformation, or embryonic lethality (McCrea and De Camilli, 2009). So far, we have mentioned PtdIns, PtdIns(4)P, and PtdIns(4,5)P_2_. There are five more phosphoinositides, most notably four important ones with phosphorylation on the 3-position (Cantley, 2002), but they will not be part of the kinetic story of this essay.

## A physiological example: M-current

**Figure 3.**
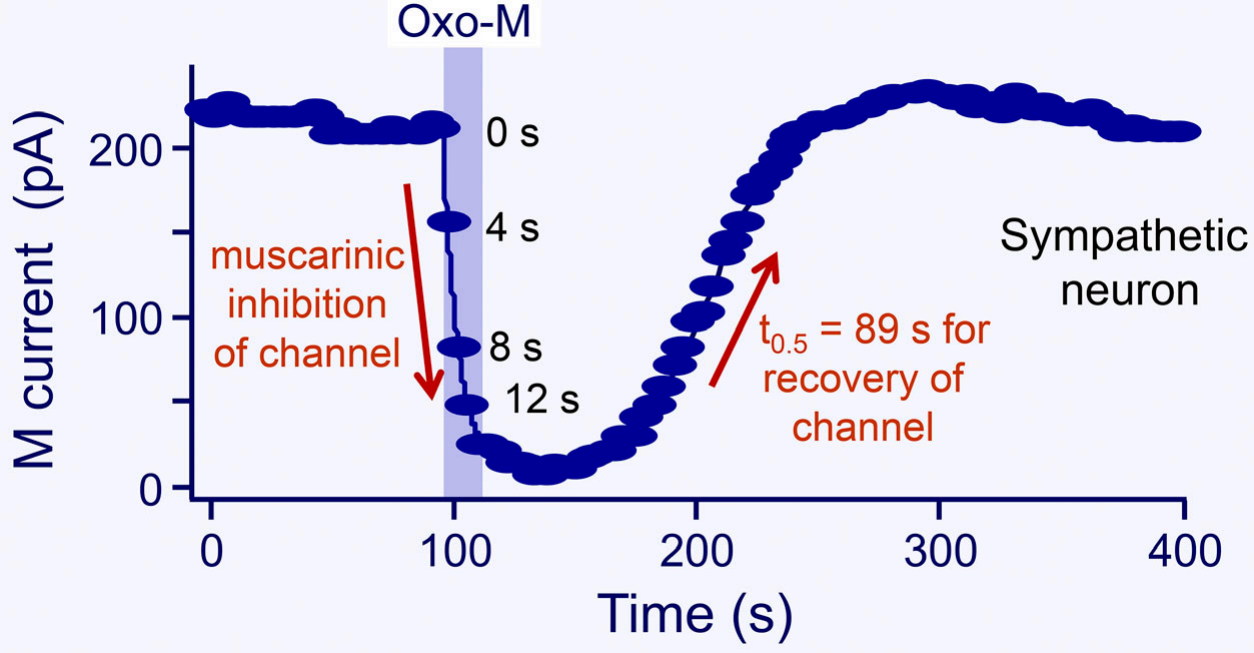
**Transient suppression of M current by a 20-s application of Oxo-M to a neuron from the rat superior cervical sympathetic ganglion.** The number labels indicate the time after the start of Oxo-M. The half-recovery time is 89 s (from Suh and Hille, 2002).

In 1980, David Brown and Paul Adams reported a chronically active K^+^ channel that they dubbed M-current (Brown and Adams, 1980). It acted as a damper that holds sympathetic ganglion cells in a state of reduced excitability. They discovered that this previously unrecognized channel could be turned off by stimulating muscarinic acetylcholine receptors (hence M-current), and then the cells became more excitable. Fig. 3 shows the muscarinic modulation of this K^+^ current in a sympathetic neuron. The current falls in a few seconds when agonist is applied, and it returns over a minute or two after agonist is removed. The same M-current was found in hippocampal neurons and elsewhere, and again it was modulated by activation of appropriate receptors (Halliwell and Adams, 1982; Marrion, 1997; Brown and Passmore, 2009). Speaking very loosely, for central neurons one might say that turning off M-current during alarm, such as a loud noise, increases excitability allowing the neurons to be more attentive and to fire in response to inputs (Brown and Passmore, 2009; Zaika et al., 2007; Kruse and Whitten, 2021). The time course of this modulation by agonists defines the window of attentiveness and contributes to arousal by muscarinic and other central pathways.

Our interest in phosphoinositides was kindled by their action on M-current. Byung-Chang Suh in our lab and Hailin Zhang in the Logothetis lab discovered that M-current (both of neurons and cells transfected with KCNQ2/3 channels, the heteromeric molecular correlate of M-current in the K_V_7 family) requires plasma membrane PtdIns(4,5)P_2_ for function (Suh and Hille, 2002; Zhang et al., 2003; Horowitz et al., 2005; Li et al., 2005; Winks et al., 2005). Suh proposed that the muscarinic modulation in Fig. 3 reflects the time course of breakdown of PtdIns(4,5)P_2_ by receptor-activated PLCβ, and that the recovery reflects the time course of resynthesis of PtdIns(4,5)P_2_ from PtdIns. There were two novel aspects to this proposal. One was that the basal concentration of PtdIns(4,5)P_2_ is low enough that it can be significantly depleted in seconds by receptor-activated PLCβ. The other was that KCNQ2/3 channel currents can be suppressed by physiological depletion of PtdIns(4,5)P_2_. Prior to that time, in pioneering work, Donald Hilgemann had discovered that K_ATP_ channels and the Na^+^–Ca^2+^ exchanger in the heart require PtdIns(4,5)P_2_ (Hilgemann and Ball, 1996; Hilgemann et al., 2001), and Diomedes Logothetis and others had generalized this requirement to many inward rectifier K^+^ channels (Huang et al., 1998; Sui et al., 1998). So a PtdIns(4,5)P_2_ requirement for KCNQ2/3 channels, although new, did not seem implausible at the time. Subsequently, >80 plasma membrane ion channels (but not all) and a number of plasma membrane transporters have been found to be PtdIns(4,5)P_2_-requiring or at least PtdIns(4,5)P_2_-sensitive (Logothetis et al., 2015; Hille et al., 2015), and crystal and cryogenic EM structures have mapped the atomic interactions with PtdIns(4,5)P_2_ in numerous channels (Whorton and MacKinnon, 2011; Sun and MacKinnon, 2020; Niu et al., 2020; Li et al., 2021; Zheng et al., 2022). Since the presence of PtdIns(4,5)P_2_ defines the plasma membrane, those channels that require PtdIns(4,5)P_2_ may be restricted from being active anywhere but in the plasma membrane (Hilgemann et al., 2001). Analogously, a requirement for PtdIns(3,5)P_2_ (which defines endolysosomes) restricts the activity of certain two-pore domain channels to the endolysosomal compartment (Wang et al., 2012). Thus, although channels and transporters traffic through many membrane compartments, many of them can be preprogrammed by lipid interactions to function only in a few.

Receptor-induced turnover of phosphoinositides measured as incorporation and release of ^32^P phosphate had been known for many decades from the work of Lowell and Mabel Hokin (Hokin and Hokin, 1955). After prolific pioneering biochemical work, Robert Michell could offer an early speculative model with PtdIns being broken down directly upon activation of specific membrane receptors to yield diacylglycerol and Ins(1)P and to open plasma membrane calcium channels (Michell, 1975). But within a few more years, it became clear that PtdIns(4,5)P_2_ was the lipid being broken down by receptor action (e.g., Michell et al., 1981; Creba et al., 1983), and that one of the products, Ins(1,4,5)P_3_, was releasing calcium from intracellular stores (Streb et al., 1983; Burgess et al., 1984). By the late 1990s, the essential enzymology of phosphoinositide metabolism was well defined, and the kinetics of PLC action were recognized as being quite fast. Thus, the time course of the PtdIns(4,5)P_2_ depletion after muscarinic, vasopressin, oxytocin, and angiotensin II application was said to be in the 15–30 s range (Creba et al, 1983; Enyedi et al, 1985; Willars et al, 1998). This was quite a new area of research for us, but as biophysicists, we thought that a more comprehensive and quantitative description of phosphoinositide dynamics could provide useful physiological context. That is the motivation of this essay.

## How fast can PtdIns(4,5)P_2_ be depleted?

To measure the speed of reactions in a living cell requires biophysical tools with adequate time resolution. The solution exchange, if needed, and the reporter probes and sampling rates must be fast. Most of our work uses single, small, isolated cells in a small, perfused microscope chamber. Biophysicists readily achieve solution-exchange times under 1 s when switching solution flow with small solenoid valves and a perfusion pipette brought within 150 μm of the small target cell. In addition, versatile optical tools have been developed to monitor phosphoinositides in living cells (Várnai et al., 2017). They are constructed as genetically expressible fusion proteins combining a fluorescent protein with an ∼100-residue protein domain derived from one of many cellular proteins that read the phosphoinositide ZIP code. Many of these affinity domains are pleckstrin homology (PH) domain sequences, of which there are at least 300 in the human genome. For PtdIns(4,5)P_2_, the most widely used reporter is a PH domain from PLCδ1 (PH_PLCδ1_; Stauffer et al., 1998; Várnai and Balla, 1998). When expressed in our cells at rest, typically 50% of the PH_PLCδ1_ reporter is bound to PtdIns(4,5)P_2_ at the cell membrane and the remainder is distributed in dynamic equilibrium in the cytoplasm. Changes in this fluorescence distribution, a direct reflection of PtdIns(4,5)P_2_ dynamics, can be followed in real-time in at least four ways: (1) by translocation in confocal microscope images (Stauffer et al., 1998; Várnai and Balla, 1998), (2) by changes in cell surface intensity with total internal reflection fluorescence microscopy, (3) by changes of Förster resonance energy transfer (FRET) when the reporters are expressed as donor-acceptor pairs of fluorescent proteins (van der Wal et al., 2001), or (4) by bioluminescence energy transfer when a luciferin PH-domain donor and an organelle-targeted fluorescent protein acceptor are coexpressed (Tóth et al., 2016). When FRET is measured with whole-cell photometry, it can follow processes happening well below the 100-ms range (Lohse et al., 2007). Luciferase-based bioluminescence energy transfer is intrinsically slower but has less background interference. Measuring ionic current can be done with microsecond resolution. Much of our work was done with easily transfected and easily patched tsA201 cells, a transformed cell line derived many decades ago from the human embryonic kidney cell line (HEK). In only a few cases has the generality of these kinetic results been tested in other types of cells and native tissue.

**Figure 4.**
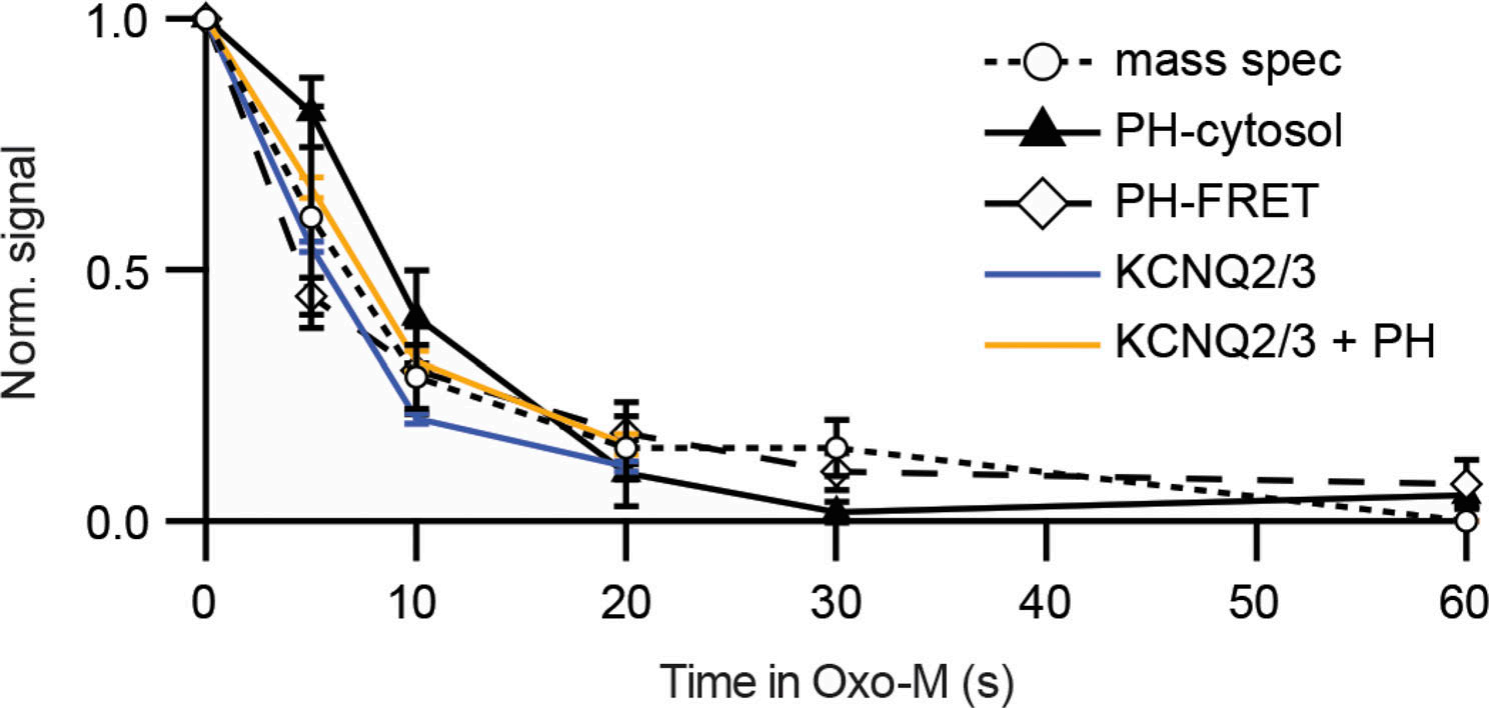
**Depletion of PtdIns(4,5)P**_**2**_ **in response to a muscarinic agonist (Oxo-M) in cell lines.** The diamonds show loss of FRET when FRET pairs PH_PLCδ1_-CFP and PH_PLCδ1_-YFP translocate from being bound near each other at the plasma membrane to being free in the cytoplasm, and the triangles show appearance of PH_PLCδ1_-YFP in the cytoplasm from confocal images (transfected tsA201 cells). Circles are PtdInsP_2_ from mass spectrometry (mass spec, in CHO-M cells stably expressing M_1_ receptors), and colored lines are amplitudes of KCNQ2/3 current (tsA201 cells; Jensen et al., 2009; from Traynor-Kaplan et al., 2017).

Our first kinetic result measures the decline of PtdIns(4,5)P_2_ following application of an agonist that activates PLCβ. Fig. 4 compares optical, electrical, and chemical measures of the PtdIns(4,5)P_2_ and KCNQ2/3 current upon addition of agonist (Traynor-Kaplan et al., 2017). The data are from a variety of experiments, but they all basically agree that depletion of PtdIns(4,5)P_2_ has a half time of 4–7 s in these cells. Said differently, when endogenous PLCβ is strongly activated, the expected half-life of any given cellular PtdIns(4,5)P_2_ molecule is only 4–7 s. These experiments are done in cell lines (tsA201 or Chinese hamster ovary [CHO] cells) transfected with muscarinic M_1_ receptors, and the muscarinic agonist is oxotremorine-M (Oxo-M) applied at a saturating concentration (10 μM). This receptor couples through G_q_ G-proteins to PLCβ. For easier comparison, the amplitudes of all responses are normalized between 1.0 and 0.0. The time courses are characterized by their half time of decay, which for the FRET of PH_PLCδ1_ pairs (containing CFP and YFP) in this experiment is 4.5 s and for confocal translocation of PH_PLCδ1_ is 8.8 s. This compares reasonably with the two curves for decay of KCNQ2/3 current, 5.7 and 7.3 s (Jensen et al., 2009), as well as with the decay of the mass spectrometry signal in CHO cells for PtdInsP_2_, 6.9 s. Perhaps the translocation signal is a little slowed down because the probe has to diffuse from the membrane into the chosen region of interest in the confocal image—an effect that would limit time resolution more strongly in much larger cells such as oocytes or when the probe also has reversible access to the nuclear compartment (Horowitz et al., 2005)—and the trace labeled KCNQ2/3 + PH is slowed because PH_PLCδ1_ probes are coexpressed and they buffer the changes of PtdIns(4,5)P_2_. In short, depletion of PtdIns(4,5)P_2_ by PLCβ can be rapid and the fall of KCNQ2/3 current follows a similar time course. It should be emphasized that although the M_1_ receptor is overexpressed in these experiments, the rest of the signaling machinery of the cell, including PLCβ itself is not overexpressed. The time course of depletion by muscarinic activation in native sympathetic neurons (Fig. 3) is about the same as that in the tsA201 cells (Fig. 4). From now on, we will take KCNQ2/3 current as a convenient direct electrophysiological indicator of plasma membrane PtdIns(4,5)P_2_. However, to be more precise, note that there are at least two PtdIns(4,5)P_2_ binding sites on each protomer of the heteromeric channel tetramer (Sun and MacKinnon, 2020; Zheng et al., 2022), and the relation of concentration to effect is not strictly linear (Telezhkin et al., 2012).

Of the methods used in Fig. 4, mass spectrometry is the most complicated since each point requires a whole dish of cells, the agonist action has to be arrested with fast pipetting of ice-cold methanol/HCl or butanol, and the acid-extracted lipids must then be derivatized (methylated) to neutralize all phosphate charges before they enter the spectrometer (Clark et al., 2011; Traynor-Kaplan et al., 2017). Using this method, it would be challenging to improve on the ∼5-s time resolution achieved. Mass spectrometry uses lipids extracted from the whole dish with no spatial resolution. As we used it, the method also would not distinguish molecules of PtdIns(4,5)P_2_ from PtdIns(3,5)P_2_ or PtdIns(3,4)P_2_ since they have the same mass. However, mass spectrometry has one distinct advantage: because it uses internal standards, it is the only method that has absolute calibration with a linear scale that starts from a known zero. The points in Fig. 4 are normalized, but our original mass spectrometry data and earlier studies with isotopes and chromatography or with HPLC (Willars et al., 1998; Horowitz et al., 2005; Traynor-Kaplan et al., 2017) show that total PtdInsP_2_ becomes depleted by 76–90% after a muscarinic stimulus. This rapid and extensive decay of the PtdInsP_2_ signal when agonist is added to the bath is a reminder that in a living cell, agonist, receptors, PLCβ, and virtually all PtdIns(4,5)P_2_ pools are quickly interacting (within 6 s) at the plasma membrane and confirm that the total cellular mass of the PtdIns(3,5)P_2_ and PtdIns(3,4)P_2_ (bisphosphates that are probably not PLCβ substrates) must be significantly lower than that of the PLCβ substrate PtdIns(4,5)P_2_.

**Figure 5.**
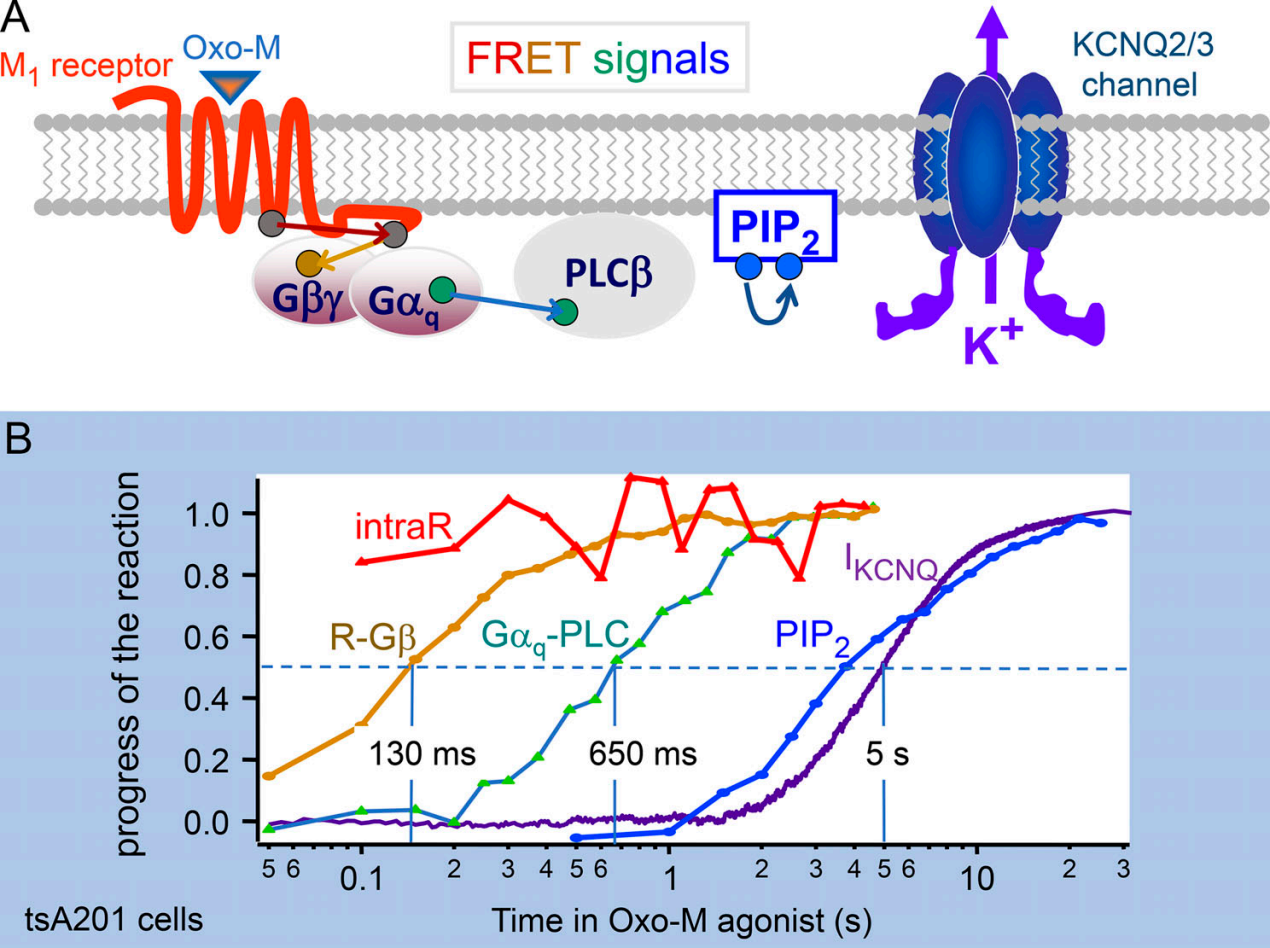
**Measuring several steps in the receptor-induced activation of PLCβ and depletion of PtdIns(4,5)P**_**2**_ **in response to a muscarinic agonist. (A)** Cartoon of the signaling pathway from muscarinic agonist to KCNQ2/3 channel. PIP_2_, PtdIns(4,5)P_2_. **(B)** YFP-CFP FRET signals, normalized to start at zero, monitor conformational changes within the receptor (intraR, intramolecular FRET between CFP and YFP labels in the receptor), receptor interaction with Gβ, Gα_q_ interaction with PLCβ, and depletion of PtdIns(4,5)P_2_. Note logarithmic time axis. Each line comes from tsA201 cells transfected with M_1_ receptors, the appropriate pair of FRET probes, or, in the case of I_KCNQ2/3_, with KCNQ2 and KCNQ3 channel subunits. Data assembled from Jensen et al. (2009).

**Figure 6.**
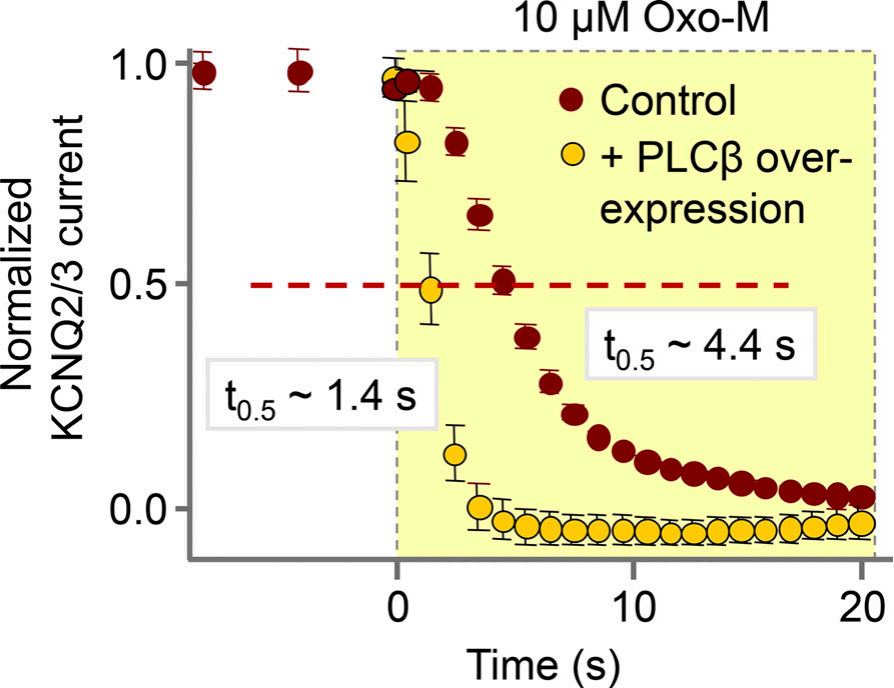
**Testing whether PLCβ is rate-limiting in the receptor-induced hydrolysis of PtdIns(4,5)P**_**2**_**.** KCNQ2/3 current suppression is three times faster in tsA201 cells overexpressing PLCβ. Half-decay times are indicated (from Jensen et al., 2009).

Why does agonist action take 6 s? Fig. 5 A outlines the intermediate steps that lead to PLCβ activation. They involve Oxo-M agonist, M_1_ receptor, the G protein G_q_, and finally activation of PLCβ by the α-subunit of G_q_. Jill Jensen (Jensen et al., 2009) was able to express CFP and YFP FRET pairs at each of the small colored ovals in the figure, for example, a receptor with a C-terminal CFP and Gβ with an N-terminal YFP. Using FRET photometry, she traced the progress of the signal initiated by applying the agonist. This style of experiment followed those of the Lohse lab (Lohse et al., 2007), who had nicely characterized the rapid signaling of adrenergic receptors to G_i_ and G_s_ using FRET. Our results plotted on a semilogarithmic axis in Fig. 5 B show that agonist modifies receptor conformation with a half time ≪100 ms, receptor speaks to Gβ in 130 ms, Gα_q_ interacts with PLCβ (transfected fluorescent PLCβ for this FRET experiment) in 650 ms, and then KCNQ2/3 current is suppressed after 5 more seconds. This leaves the conclusion that turning on PLCβ takes ∼1 s, and that most of the several-second time to modulate KCNQ2/3 channels is used for activated PLCβ enzymes to complete the hydrolysis of the pools of plasma membrane PtdIns(4,5)P_2_. As confirmation that endogenous PLCβ is rate-limiting in channel modulation, Fig. 6 shows that the half time of KCNQ2/3 current suppression is shortened to 1.4 s if endogenous PLCβ in the cell is supplemented by overexpressing an exogenous PLCβ.

Similar experiments have been done in a few mammalian neurons. As we said already, for superior cervical ganglion neurons, the half time for muscarinic inhibition of M-currents is the same as in tsA201 cells (Fig. 3; Suh and Hille, 2002; Kruse et al., 2016). For endogenous muscarinic or metabotropic glutamate receptor–induced translocation of PH_PLCδ1_ in hippocampal neurons, the half times are ∼12 and 20 s (de la Cruz et al, 2022b). In dorsal root ganglion neurons, the half time for translocation of tubby (another PtdIns[4,5]P_2_ probe) in response to capsaicin is ∼8 s (Lukacs et al., 2013). The estimate is approximate since samples were taken only every 8 s. Here, capsaicin allows Ca^2+^ entry via transient receptor potential (TRP) channels, activating Ca^2+^-sensitive PLCδ rather than PLCβ. For an extreme example of physiological adaptation for speed, we mention the eye of the fly where PLCβ activated by light can initiate channel opening (*Drosophila* TRP and *Drosophila* TRP-like) within 20 ms (Hardie and Juusola, 2015)! Presumably there, the geometry and expression levels have been optimized to enable the channels to sense lipid perturbations after only a few catalytic cycles of PLC.

**Figure 7.**
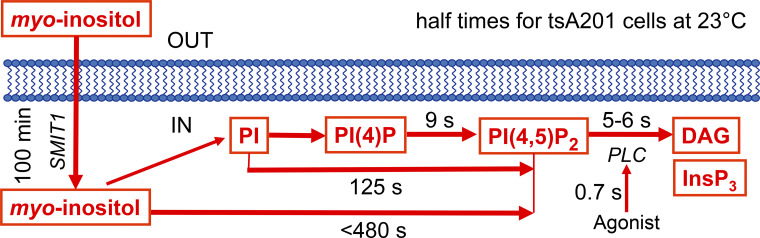
**Summary of half times for PtdIns(4,5)P**_**2**_ **synthesis and breakdown.** The diagram represents some important steps in phosphoinositide metabolism—the PI cycle—and the measured half times for segments we could study in living tsA201 cells at room temperature as explained in the text. Everything might be three to four times faster at 37°C. DAG, diacylglycerol; PI, PtdIns; PI(4)P, PtdIns(4)P; PI(4,5)P_2_, PtdIns(4,5)P_2_.

This essay is primarily about relevant and measurable metabolic half times of phosphoinositide metabolism in living cells—usually tsA cells. For ease of keeping track of these values as they are extracted from experiments, they are summarized in the kinetic diagram in Fig. 7. There we record that it takes 0.7 s for the agonist to activate PLCβ, which in turn takes 5–6 s to deplete PtdIns(4,5)P_2_.

## How fast can PtdIns(4,5)P_2_ be resynthesized?

**Figure 8.**
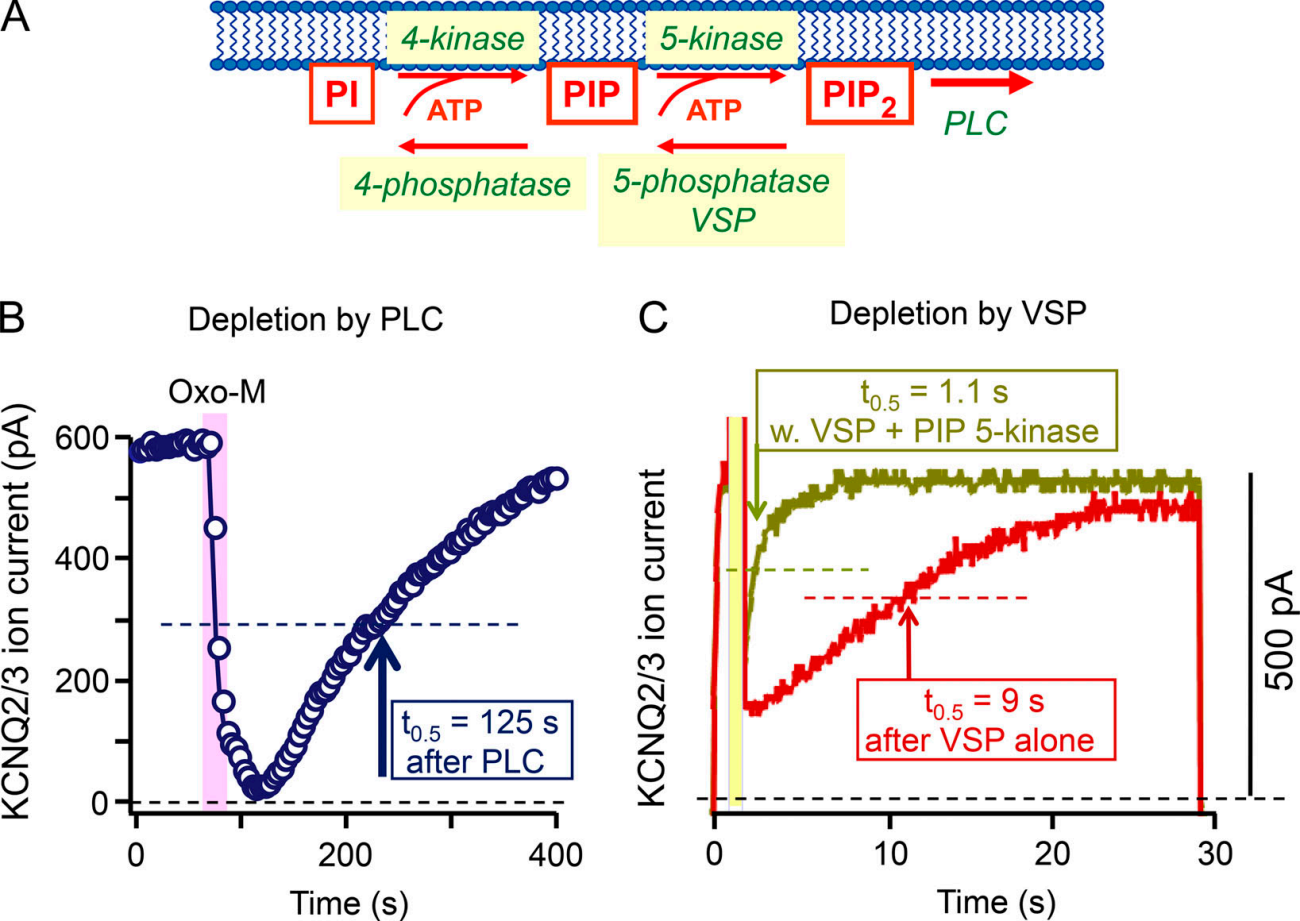
**Recovery of PtdIns(4,5)P**_**2**_ **after depletion.** KCNQ2/3 current suppression and recovery are recorded to reflect depletion and recovery of PtdIns(4,5)P_2_. Recovery half times are indicated. **(A)** Diagram of PtdIns(4,5)P_2_ resynthesis showing also phosphatases and the voltage-sensitive VSP enzyme. **(B)** Depletion and recovery after a short application (25 s) of Oxo-M agonist (10 μM). The experiment is similar to that in Fig. 3 except that rather than using a neuron with endogenous receptors and channels, this one uses tsA201 cells transfected with M_1_ receptors and KCNQ2 and KCNQ3 channel subunits. PIP_2_, PtdIns(4,5)P_2_. From Horowitz et al. (2005). **(C)** A tsA201 cell transfected with channels and VSP (no receptors) is depolarized in a three-step protocol starting at 0 s. The first 1-s step to −20 mV activates KCNQ2/3 current, the next 1-s step to 100 mV activates VSP briefly, and the third step to −20 mV monitors current recovery during the resynthesis of PtdIns(4,5)P_2_. The olive trace shows a cell that had also been transfected with PIP 5-kinase. From Falkenburger et al. (2010a).

We have been considering the kinetics of rapid PtdIns(4,5)P_2_ breakdown, and we now turn to its restoration (Figs. 3 and 8 A). In the neuron example, if we can say loosely that lipid breakdown enables attention, then lipid restoration would close that window of attention. Fig. 8 B shows recovery of KCNQ2/3 current with a half time of 125 s after brief activation of muscarinic receptors in a tsA201 cell (Suh and Hille, 2002; Horowitz et al., 2005). In the sympathetic neuron shown in Fig. 3, recovery is 30–60% faster (Suh and Hille, 2002; Kruse et al., 2016), and in hippocampal neurons, translocation of PH_PLCδ1_ is fivefold faster (de la Cruz et al., 2022b). In the hippocampus, recovery is speeded by having an enlarged pool of PtdIns(4)P (de la Cruz et al., 2022b). It would be interesting to determine how much the lipid pools and the recovery are optimized to match the different roles of different cell types.

What are the underlying molecular events? The immediate precursor of PtdIns(4,5)P_2_ is PtdIns(4)P. However, in agreement with Willars et al. (1998) and Balla et al. (2008), working with other cultured cell lines (human neuroblastoma SH-SY5Y and embryonic kidney HEK293), our mass spectrometry experiments in tsA201 cells showed that PtdInsP pools are strongly depleted during application of agonist (Traynor-Kaplan et al., 2017), whereas the much larger total PtdIns pool is little affected. Therefore, resynthesis of PtdIns(4,5)P_2_ has to start from the still virtually intact pool of PtdIns and proceed in two phosphorylation steps via PI 4-kinase and PI4P 5-kinase reactions. (Fig.8 A). To isolate one of these two steps, we took advantage of an enzymatic tool, a voltage-sensing lipid 5-phosphatase (VSP; Murata et al., 2005), that allowed us to convert much of the PtdIns(4,5)P_2_ pool abruptly to PtdIns(4)P at the plasma membrane by a 1-s, very large (100 mV) depolarizing voltage pulse. The lipid recovery from this perturbation would reflect the endogenous PI4P 5-kinase enzymes acting alone at the plasma membrane to convert the artificially enlarged PtdIns(4)P pool back to PtdIns(4,5)P_2_. The red trace in Fig. 8 C shows that this recovery from VSP activation is >10 times faster than the recovery after receptor and PLCβ activation in Fig. 8 B. Thus, the much slower overall recovery in Fig. 8 B indicates that the PI 4-kinase step is normally slow and rate-limiting. Confirmation of the faster action of PI4P 5-kinase is shown by the olive trace in Fig. 8 C, where overexpression of more PI4P 5-kinase enzyme speeds recovery from VSP by an additional eightfold. The half times for resynthesis of PtdIns(4,5)P_2_ from PtdIns(4)P (9 s) and from PtdIns (125 s) are summarized in Fig. 7. In contrast, resynthesis of PtdIns(4,5)P_2_ in hippocampal neurons proceeds directly from their larger pool of PtdIns(4)P, and so is more rapid (25 s; de la Cruz et al., 2022b).

## Speed of our tools revisited

Being voltage-activated, the VSP enzyme offers the fastest perturbation of phosphoinositides in our toolset with a half time for PtdIns(4,5)P_2_ depletion of <100 ms. With VSP overexpressed, a strong depolarizing pulse (to +100 or +110 mV) suppresses currents in KCNQ2/3, Ca_V_1.3, and Ca_V_2.2 channels with half times of ∼100 ms (Falkenburger et al., 2010b; Suh et al., 2010). Similarly, a 300-ms pulse decreases PH_PLCδ1_ FRET by 90% of the maximum (Falkenburger et al., 2010b), and the reported in vitro half-life of the PH_PLCδ1_–PtdIns(4,5)P_2_ complex is 46 ms (Pilling et al., 2011). Collectively, such data allow us to say that the complexes between channels and PtdIns(4,5)P_2_ and between PH_PLCδ1_ and PtdIns(4,5)P_2_ are rapidly formed and short-lived with half times ≪100 ms, making channels and probes good reporters of “instantaneous” levels of PtdIns(4,5)P_2_. Thus, one should not think that channels or PH-domain probes hold on to one lipid molecule for long times. There is continuous exchange in these relatively low-affinity interactions, which for PH_PLCδ1_ with PtdIns(4,5)P_2_ is reported to have a dissociation constant of 0.15–3 μM (Lemmon et al., 1995; Hirose et al., 1999; Pilling et al., 2011).

## Another physiological example: Vascular control

Mark Nelson has pointed out that numerous key ion channels in vascular smooth muscle and endothelial cells are sensitive to levels of PtdIns(4,5)P_2_. The channels contribute both to the resting membrane potential in these electrically coupled cells and to entry of Ca^2+^ ions from the extracellular space and thus to vascular tone. For example, in regulation of cerebral blood flow (Harraz et al., 2020), hyperpolarization at the level of the capillary endothelium can be communicated electrically to the upstream arteriolar endothelium and to the enveloping arteriolar smooth muscle, initiating relaxation and greater blood flow. This retrograde electrical signaling requires hyperpolarizing K^+^ channels like Kir2.1, a PtdIns(4,5)P_2_-dependent channel, and it fails when input from G_q_-coupled receptors (muscarinic, adrenergic, angiotensin II, vasopressin, etc.) depletes PtdIns(4,5)P_2_. Thus, these agonists can induce vasoconstriction, decrease capillary perfusion, and raise blood pressure at least in part by their action on PtdIns(4,5)P_2_ levels and consequently, ion channels. The effects of PtdIns(4,5)P_2_ depletion would be occurring in parallel with already known signaling from Ins(1,4,5)P_3_ and diacylglycerol. Because there are many channel types and many vascular beds, this vascular story is likely to be complex, and the unifying involvement of PtdIns(4,5)P_2_ is only beginning to be recognized as emphasized by the review of Harraz et al. (2020).

## How quickly does *myo*-inositol enter the phosphoinositide cycle?

Once PtdIns(4,5)P_2_ is broken down by PLCβ to Ins(1,4,5)P_3_ and diacylglycerol, the diffusible Ins(1,4,5)P_3_ can be recycled. Three dephosphorylation steps yield the *myo*-inositol precursor that will be rejoined with CDP-diacylglycerol to resynthesize PtdIns, as in Fig. 2. We were surprised to find that the size of cellular PtdIns(4,5)P_2_ and PtdIns(4)P pools also can be markedly increased merely by increasing the supply of *myo*-inositol from the culture medium (Dai et al., 2016). Gucan Dai transfected cells with SMIT1, the sodium-coupled *myo*-inositol cotransporter, and then increased the *myo*-inositol concentration in the culture medium from 40 to 140 μM to test for induced changes in phosphoinositide pools by mass spectrometry. Remarkably, after overnight incubation, the PtdInsP_2_ and PtdInsP pools both had grown from a normalized value of 1.0 to a value of 2.7, whereas the total PtdIns pool showed no detected change. If the cells were exposed to increased medium *myo*-inositol without SMIT1, the changes were intermediate. The increase of PtdIns(4,5)P_2_ also was manifested in several aspects of the physiology of the cells when they were additionally transfected with KCNQ2/3 channels. The voltage-dependence of the activation and deactivation gating kinetics of the channels was changed, the time it took for PLCβ to suppress KCNQ2/3 current by depleting PtdIns(4,5)P_2_ was lengthened, and the amounts of Ins(1,4,5)P_3_ and intracellular Ca^2+^ released by agonists were increased (Dai et al., 2016). For superior cervical ganglion neurons, which abundantly express native KCNQ2/3 channels, similar overnight *myo*-inositol supplementation attenuated the action potential firing, consistent with a stronger KCNQ2/3 brake on firing when more PtdIns(4,5)P_2_ is produced. Such measurable functional changes provided a way to determine the kinetics of entry of *myo*-inositol into the PI cycle. Using the real-time electrophysiological criteria, if SMIT1 transporters were overexpressed, the half time of PtdIns(4,5)P_2_ rise was 100 min when *myo*-inositol was added to the bathing medium, and it was shortened to 8 min when *myo*-inositol was added directly into the cytoplasm by diffusion from the whole-cell pipette (Dai et al., 2016). Included in the 8-min half time is the time for *myo*-inositol to diffuse out of the pipette. Again, these numbers measured in tsA201 cells are reported in Fig. 7. Evidently, phosphoinositide pools, the excitability of at least peripheral neurons, and the function of membrane proteins would be potentially sensitive to dietary intake of *myo*-inositol, an effect that might take only an hour or two to develop. Interestingly, *myo*-inositol is also an osmolyte, and SMIT1 participates in osmotic homeostasis. SMIT1 expression increases in response to extracellular hypertonicity, elevating *myo*-inositol levels inside of the cell adaptively during osmotic stress. Simultaneously the accumulation of *myo*-inositol in response to hypertonicity should regulate ion channel activities, intracellular calcium signaling, and neuronal excitability. Indeed, in the presence of the extra *myo*-inositol, overnight hypertonicity treatment (+150 mOsm raffinose) quantitatively recapitulated nearly all the measurable effects produced by SMIT1 overexpression (Dai et al., 2016). Thus, hypertonic responses would be accompanied by changes of cell excitability.

## Regulation revealed by longer agonist applications

When monitoring the products of PtdIns(4,5)P_2_ hydrolysis (Fig. 2) after receptor activation, we observed major regulation of PtdIns(4,5)P_2_ synthesis. In the 1980s, through the work of Michael Berridge, Robin Irvine, and James Putney (Streb et al., 1983; Burgess et al., 1984) and of Yasutomi Nishizuka (Kishimoto et al., 1980; Suh et al., 1988), signaling roles for receptor-activated PLC came to widespread attention. Especially, interesting for cell physiologists were rapid mobilization of intracellular Ca^2+^ by Ins(1,4,5)P_3_, the soluble product of PtdIns(4,5)P_2_ hydrolysis (Streb et al., 1983; Burgess et al., 1984), and the identification of membrane diacylglycerol as the stimulus for protein kinase C (Kishimoto et al., 1980). Thus, Ins(1,4,5)P_3_ and diacylglycerol became recognized as powerful phosphoinositide-derived second messengers mediating receptor signals more than a decade before PtdIns(4,5)P_2_ itself was recognized as a second messenger. At that time, PtdIns(4,5)P_2_ was just considered the lipid precursor of important signals.

**Figure 9.**
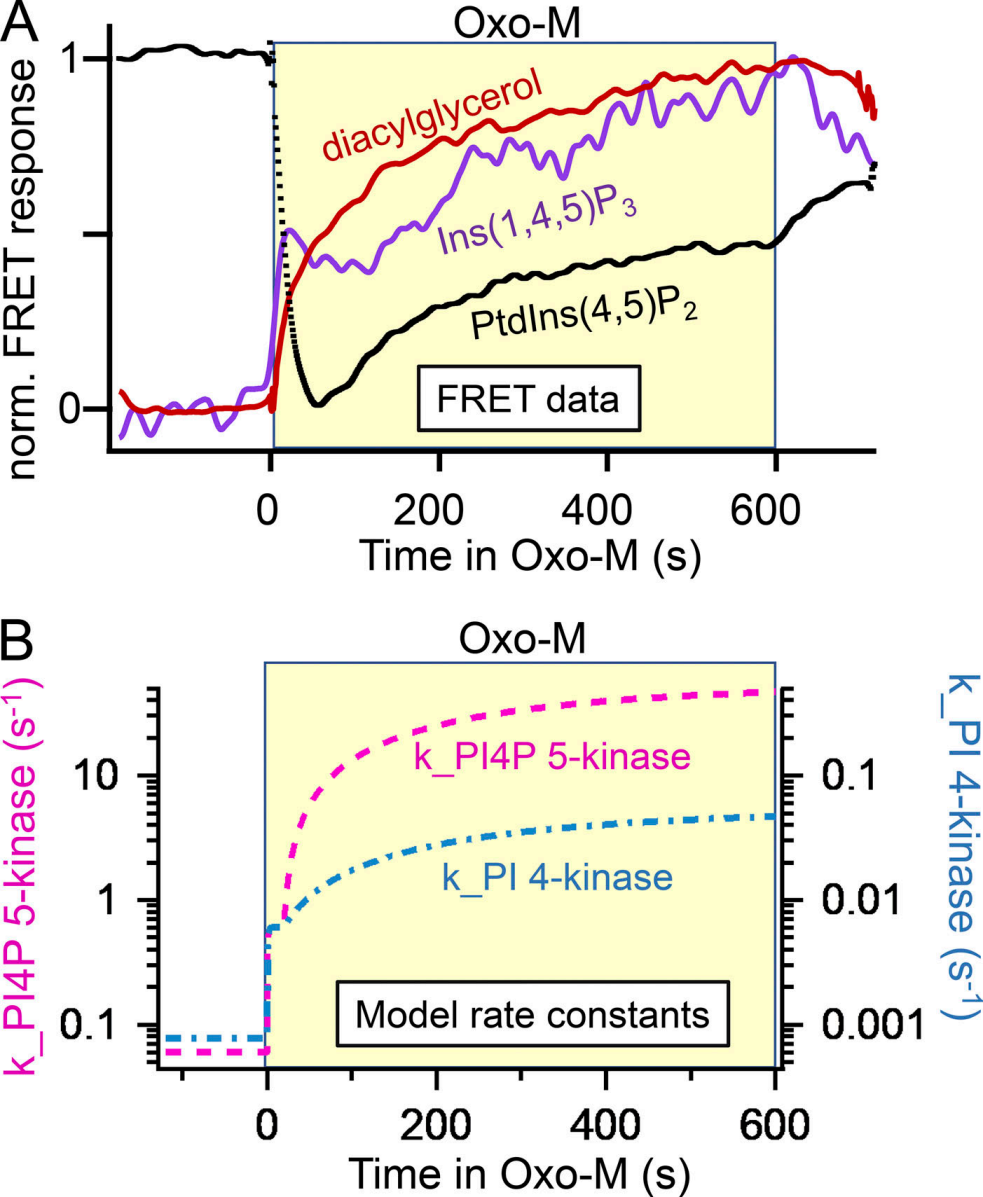
**Generation of Ins(1,4,5)P**_**3**_ **and diacylglycerol during prolonged agonist application—and the simultaneous regeneration of PtdIns(4,5)P**_**2**_**. (A)** Representative normalized FRET traces from three cells using C1A domain (diacylglycerol), LIBRAvIII (Ins[1,4,5]P_3_), and PH_PLCδ1_ (PtdIns[4,5]P_2_) probes. **(B)** Time courses of effective rate constants of lipid kinases from the mathematical model showing upregulation during agonist action. Data and model modified from Myeong et al. (2020).

The time courses of production of the Ins(1,4,5)P_3_ and diacylglycerol second messengers and of depletion of PtdIns(4,5)P_2_ during a 10-min application of agonist are measured with FRET probes in Fig. 9 A (Myeong et al., 2020). This long exposure to agonist is not exactly like any physiological situation but, nevertheless, uncovers interesting kinetic processes. Our results parallel those of Tóth et al. (2016). As expected, both second messengers appear in an initial burst while their precursor PtdIns(4,5)P_2_ falls abruptly. The production of Ins(1,4,5)P_3_ and diacylglycerol is so intense that their lipid precursor becomes depleted; yet, despite this initial depletion, the cell continues to produce more InsP_3_ and more diacylglycerol, and, remarkably, despite the continued activity of PLCβ, the cellular PtdIns(4,5)P_2_ actually begins to rise again, a growth we called regeneration (Myeong et al., 2020). The regeneration could be cut short if the recycling of *myo*-inositol by dephosphorylation of Ins(1,4,5)P_3_ was inhibited by adding lithium to the medium (see Fig. 2). Although the breakdown of PtdIns(4,5)P_2_ and the production of second messengers are obligately coupled one-for-one, after some seconds, the three time courses deviate. These nonintuitive details needed further kinetic explanation.

Over the last two decades, each of our kinetic studies was accompanied by kinetic modeling that tested our interpretations. Thus, all the steps in Figs. 2, 5 A, and 7 and many more have been represented by chemical kinetic equations in our constantly improving, publicly available, kinetic model of phosphoinositide metabolism (Suh et al., 2004; Horowitz et al., 2005; Falkenburger et al., 2010a; Falkenburger et al., 2010b, Falkenburger et al., 2013; Dickson et al., 2013; Jung et al., 2016; Myeong et al., 2020). The model was developed in the Virtual Cell simulation environment (Cowan et al., 2012) following the pioneering example of Xu et al. (2003).

The modeling provided an explanation of the traces in Fig. 9 A and revealed new mechanisms (Myeong et al., 2020). Although they are generated synchronously, the time courses for Ins(1,4,5)P_3_ and diacylglycerol differ because their downstream metabolism and their eventual cellular redistribution differ. The half-life of Ins(1,4,5)P_3_ is short, on the order of 5–30 s (Sims and Allbritton, 1998; Fink et al., 1999; Xu et al., 2003; Falkenburger et al., 2013; Tóth et al., 2016). This rapid breakdown means that the Ins(1,4,5)P_3_ trace is nearly proportional to the instantaneous rate of production rather than representing the cumulative amount produced. Ins(1,4,5)P_3_ production shows an initial burst that slows only a little as PtdIns(4,5)P_2_ becomes strongly depleted, but then gradually picks up again reaching an even higher level than in the initial burst. On the other hand, the effective half-life for diacylglycerol molecules is one or several minutes (Suh and Hille, 2006; Jung et al., 2016; Schuhmacher et al., 2020), and this slower breakdown means that the diacylglycerol trace is smoothened and reports the cumulative amount produced over the previous 2–4 min. Once again, the diacylglycerol trace shows that production in the last few minutes significantly exceeds that in the first few minutes of agonist action.

Modeling revealed that there is no way to explain the later continued rise of Ins(1,4,5)P_3_, diacylglycerol, and their precursor PtdIns(4,5)P_2_ without proposing that the synthesis of PtdIns(4,5)P_2_ is gradually but dramatically accelerating throughout the 10-min period of agonist (Falkenburger et al., 2013; Myeong et al., 2020). This was modeled by gradually increasing the effective rate constants for the PI 4-kinase (see also Tóth et al., 2016) and the PIP 5-kinase enzymes as described in Fig. 9 B. In an early boost, some of the upregulation occurs in the first few seconds of agonist application (Xu et al., 2003; Falkenburger et al., 2013), but much more happens over the following minutes. Compared to their basal values, the effective rate constants for PI 4-kinase and PIP 5-kinase increase in these several stages eventually by as much as 40- and 500-fold, respectively, during 10 min of agonist exposure, a strikingly enormous dynamic range of upregulation (Myeong et al., 2020). It should be noted that M_1_ muscarinic receptors desensitize unusually slowly compared with many other G-protein coupled receptors, and that the events in Fig. 9 A could not be modeled on the assumption that they would be due to strong receptor desensitization. For example, in that case, production of Ins(1,4,5)P_3_ and diacylglycerol would stop rather than speeding up. Previously, Xu et al. (2003) had done similar experiments stimulating rapidly desensitizing bradykinin receptors of neuroblastoma cells. Their modeling with the just-developed Virtual Cell system had also concluded that PI 4-kinase and PI4P 5-kinase increased abruptly ∼5- and 20-fold, respectively, upon bradykinin addition. However, then the kinase rates decayed very quickly back to basal (time constant = 1 s) in their system with fast desensitizing bradykinin receptors. Similarly, Tóth et al. (2016) had shown increases of PtdIns(4)P synthesis with moderate stimulation of epidermal growth factor or muscarinic M_3_ receptors in cultured cell lines, an effect they could attribute to feedback from protein kinase C.

Although our experiments gave quantitative details of the magnitude and time courses of effective upregulation of two lipid kinase steps, they did not reveal the underlying mechanisms. What signals and what molecular events lead to upregulation? Is the catalytic turnover number of individual kinases increased, are the kinases recruited and reorganized at appropriate phosphoinositide pools to make them more efficient, or is the mobilization and delivery of their substrates and products from other lipid pools more effective? These ideas remain for future elaboration, but there are already suggestive leads that we can now describe.

## Examples of metabolic control

Consider several provocative mechanistic studies that tie receptor activation to signaling pathways including regulation of lipid kinases. We think of them as offering possible mechanisms for the phosphoinositide kinase regulation that we described during long agonist application. While acknowledging that protein phosphorylation and allosteric regulation by substrates and products are known, powerful, and likely regulatory mechanisms in phosphoinositide metabolism, to broaden this discussion, we call attention to several other potential mechanisms starting with receptor-induced scaffolding of lipid kinase enzymes. Again, we aim for kinetic information.

### The arrestin scaffold

PtdIns(4,5)P_2_ is needed in several steps of receptor endocytosis mediated by the scaffolds β-arrestin and clathrin. Classical experiments with β-adrenergic receptors defined the endocytic mechanism of receptor desensitization at the plasma membrane. They showed phosphorylation of agonist-occupied adrenergic receptors by G-protein coupled receptor kinase, followed by binding of the phosphorylated and ligand-occupied receptor with β-arrestin (Luttrell et al., 2001; Shenoy and Lefkowitz, 2011), formation of clathrin-coated pits guided by membrane-bound adapter protein 2 (AP2; Antonescu et al., 2011; Kadlecova et al., 2017), and clathrin-mediated endocytosis of the receptor–β-arrestin complexes in vesicles that are pinched off by the GTPase dynamin (Hinshaw and Schmid, 1995; Roux et al., 2006). PtdIns(4,5)P_2_ binds to and helps to localize or activate at least four of these proteins: G-protein coupled receptor kinase, β-arrestin, AP2, and dynamin, thus concentrating the components specifically at the plasma membrane after receptor activation. Since the site for stable binding of β-arrestin on the receptor core overlaps with that for G-proteins, the stable binding mode of β-arrestin suffices to occlude coupling via G proteins, and then, endocytosis removes receptors from the cell surface. Both AP2 and β-arrestin are scaffolds that bring together and activate many signaling components. Within a couple of minutes, they upregulate local PtdIns(4,5)P_2_ synthesis by inter alia recruiting PIP 5-kinase I to the membrane (Krauss et al., 2006; Nelson et al., 2008). In an interesting illustration of phosphoinositide ZIP code function, clathrin-coated pits are also used for similar budding of secretory vesicles from the trans-Golgi network, a membrane lacking PtdIns(4,5)P_2_ but marked by PtdIns(4)P (Wang et al., 2003). There, the clathrin adaptor is AP-1, which localizes with and is activated by PtdIns(4)P; this complex recruits a PtdIns(4)P-synthesizing enzyme PI 4-kinase (Wang et al., 2003; Haucke, 2005; Wieffer et al., 2013). Thus, both in endocytosis and in budding from the Golgi, there is local upregulation of synthesis of an appropriate essential phosphoinositide lipid by recruitment of lipid kinases on protein scaffolds.

We have studied the kinetics of two types of signaling through β-arrestin, one involving rapidly desensitizing protease-activated PAR2 receptors and the other involving weakly desensitizing muscarinic M_1_ receptors. Both receptors couple to G_q_ and PLCβ and initiate powerful depletion of PtdIns(4,5)P_2_. We used FRET and single-molecule imaging in these experiments. For the PAR2 receptor, there was an apparent paradox that a receptor that activates PtdIns(4,5)P_2_ breakdown strongly is nevertheless rapidly internalized by PtdIns(4,5)P_2_-dependent clathrin-mediated endocytosis. As expected, our PH_PLCδ1_ probe showed in tsA201 cells that PtdIns(4,5)P_2_ is very strongly depleted upon PAR2 agonist application with a familiar half time of 6 s (endogenous PLCβ is again limiting); however then, with agonist still present, the PtdIns(4,5)P_2_ nevertheless recovers with a half time of 60 s, and by 90 s it has well overshot the basal level (Jung et al., 2016, Jung et al., 2021). Thus, unexpectedly, PAR2 receptor activation actually increases PtdIns(4,5)P_2_ after a minute. In the same time, the receptor binds stably to β-arrestin (half time ∼50 s), the receptor–arrestin complexes can be seen mingling into clathrin-coated pits, and the PtdIns(4,5)P_2_-synthesizing enzyme PIP 5-kinase is seen reversibly interacting with the complexes. In short, PtdIns(4,5)P_2_ is quickly depleted, then receptors are being desensitized in 1 min, and simultaneously an overshooting restoration of PtdIns(4,5)P_2_ by recruitment of PIP 5-kinase allows endocytosis to proceed. If β-arrestin is mutated in its binding site for PtdIns(4,5)P_2_ or is knocked down with small interfering RNA (siRNA), the recruitment of β-arrestin from the cytoplasm, the resynthesis of PtdIns(4,5)P_2_, and the internalization of PAR2 receptors are all compromised (Jung et al., 2021).

In another scaffolding role, β-arrestin can assemble the entire extracellular signal-regulated kinase (ERK/MAPK [mitogen-activated protein kinase]) cascade. It has binding sites for sequentially acting cRaf (MAPK kinase kinase), MEK (MAPK kinase), and ERK (MAPK) that mediate a signaling cascade for cell proliferation, cell migration, and actin dynamics after activation of G-protein coupled receptors (Luttrell et al., 2001; Shenoy and Lefkowitz, 2011; Jung et al., 2017; Kim et al., 2022). In our work with optical probes and single-molecule total internal reflection fluorescence, we saw a recruitment and loose (reversible in seconds) association of β-arrestin with M_1_ receptors developing in ∼5 s (half time) of agonist application, followed by a stable (reversible only in many minutes) receptor association with β-arrestin developing in 10 min, and phosphorylation of ERK accumulating slowly over 5–10 min (Jung et al., 2017; Jung and Hille, 2019). Modeling suggested concomitant phosphorylation of the M_1_ receptor developing in 5 s. Despite the formation of about 30% stable β-arrestin–receptor complexes, almost none of the M_1_ receptors are internalized in the first 10 min, but the ERK/MAPK pathway is strongly activated and in parallel with it, presumably, the activities of PI 4-kinase and of PIP 5-kinase gradually become greatly accelerated, speeding the regeneration of more PtdIns(4,5)P_2_ as described in Fig. 9.

### Dishevelled and IQGAP scaffolds

Due to the similarity in assembling signaling pathways after receptor activation, we describe two other examples of scaffolds. We start with dishevelled, a scaffold of the Wnt signaling pathway. Both canonical and non-canonical Wnt signaling begin with an extracellular Wnt typically binding to the frizzled receptor, which recruits cytoplasmic dishevelled to the plasma membrane. In the usual descriptions, membrane dishevelled then binds axin, GSK3, and β-catenin for the canonical Wnt pathway and DAAM1, Rac, and Rho for the non-canonical pathway (Veeman et al., 2003). Among 38 proteins listed to interact with dishevelled are the 6 above but also transcription factors, protein phosphatases and kinases, β-arrestin, and another scaffold that we consider later, IQGAP (IQ motif-containing GTPase activating protein; Sharma et al., 2018). In addition, dishevelled colocalizes two phosphoinositide-lipid kinases. One study in HEK293T cells showed by co-immunoprecipitation physical association among PI4K type IIα, PIP5K, and dishevelled during Wnt3a signaling, accompanied by an increase in PtdIns(4,5)P_2_ (Qin et al., 2009). Similarly, we found a rise of PtdIns(4,5)P_2_ in tsA201 cells transfected with the Ror2 receptor (a tyrosine-kinase, non-canonical Wnt receptor) stimulated by Wnt 5a signaling (de la Cruz et al., 2022a). We saw that with Wnt application, dishevelled 3 coordinates PI 4-kinase IIIα and PIP 5-kinase Iγ, bringing them close together (more FRET). It recruits at least the 5-kinase to the plasma membrane and stimulates net PtdIns(4,5)P_2_ synthesis. The rise of PtdIns(4,5)P_2_ also could be induced simply by overexpressing Ror2 or dishevelled 3, or by overexpressing both PI4KIIIα and PIP5KIγ together. In agreement, PtdIns(4,5)P_2_ is decreased when dishevelled is knocked down by siRNA. Thus, especially during Wnt stimulation, dishevelled seems to upregulate PtdIns(4,5)P_2_ production by coordinating a sequential two-step synthesis from PtdIns to PtdIns(4,5)P_2_.

IQGAPs are abundant scaffolds that interact with >100 other proteins; they serve in receptor signaling through arrestin, frizzled, tyrosine kinase receptors, and integrins (Smith et al., 2015). Like β-arrestin, they can assemble the entire MAPK pathway, but of most interest here is their interaction with phosphoinositide signaling. IQGAP1 can coordinate the synthesis and signaling of PtdIns(3,4,5)P_3_ all the way from PtdIns (Choi et al., 2016; Yerramilli et al., 2022). Thus, it assembles PI 4-kinase IIIα, PI4P 5-kinase Iα, and PIP_2_ 3-kinase, as well as the PtdIns(3,4,5)P_3_-effectors PKD1 and Akt onto a single platform. PtdIns can enter the assembly line, efficiently delivering PtdIns(3,4,5)P_3_ directly to the two PtdIns(3,4,5)P_3_-stimulated kinases. Tests show that when the PtdIns(4)P and PtdIns(4,5)P_2_ intermediate lipids are formed transiently in the assembly line, they are not accessible to cytoplasmic probes (Choi et al., 2016). Instead, they are channeled right on to the next enzyme. Although the immediate precursor to PtdIns(3,4,5)P_3_ is PtdIns(4,5)P_2_, this synthesis can take place starting with PtdIns in membranes that have no free PtdIns(4,5)P_2_.

In sum, these examples, where arrestin, dishevelled, and IQGAP accelerate PtdIns(4,5)P_2_ production by colocalization of lipid kinases, suggest possible mechanisms for the dramatic upregulation of PtdIns(4,5)P_2_ production (regeneration) that we saw during long muscarinic agonist action.

### Topological organization and transport

Beyond scaffolding, we need to consider possible regulation of lipid transport and segregation. The cell has many membranes, each with its own complement of phosphoinositides and served by specific isotypes of lipid kinases and phosphatases (Fig. 2; Nakatsu et al., 2012; Balla, 2013; Pemberton et al., 2020; Zewe et al., 2020). Even at the plasma membrane, phosphoinositide metabolism is segregated between cholesterol-rich, liquid-ordered “raft” domains and cholesterol-poor, liquid disordered “non-raft” domains (Myeong et al., 2021). Using specific fluorescent markers that segregate into either raft or non-raft domains for kinetic analysis, the PtdIns(4,5)P_2_ depletion and its recovery after receptor activation were faster in the lipid-raft than in the non-raft domains (Myeong et al., 2021). The PtdIns(4,5)P_2_-sensitive KCNQ2/3 channels can be recruited to lipid-raft domains by other proteins including palmitoylated β-secretase involved in the Alzheimer’s disease mechanism (Dai, 2022). While we have been focused on the plasma membrane, when we speak of PtdIns(4,5)P_2_ synthesis, we must consider phosphoinositide precursors and enzyme isotypes in many membranes. Their transport and activity may be rate-limiting, and resolving them and representing them well in models will be challenging. For example, if an engineered 4-phosphatase enzyme (a mini-sac1) is chemically recruited using dimerization induced by rapamycin to the trans-Golgi apparatus to deplete PtdIns(4)P there, the KCNQ2/3 current of the plasma membrane falls by 30% with a half time of 40 s (Dickson et al., 2014), emphasizing rapid lipid traffic and conversion between the Golgi and the plasma membrane.

For transport, older literature considered bulk inter-compartmental lipid transfer by membrane fusion and fission, whereas newer literature emphasizes non-vesicular lipid molecule transfers through tunnel-like or cup-like phosphoinositide transfer proteins at junctional complexes between the different membranes (Reinisch and Prinz, 2021). Lipid transfer proteins, such as Nir2, extended synaptotagmins, OSBP (oxysterol-binding protein), and ORP5/8/10 (OSBP-related protein) transfer or exchange phosphoinositides across ER–plasma membrane junctions (e.g., Kawasaki et al., 2022). Junctions containing Nir2 form after G_q_-coupled receptor activation when Nir2 recognizes diacylglycerol and phosphatidic acid on the plasma membrane; there they transfer PtdIns from the ER in exchange for plasma membrane phosphatidic acid (Kim et al., 2016). Indeed, Nir2 is essential for the resynthesis of PtdIns(4,5)P_2_ after depletion. Such lipid transport proteins have not been a focus of quantitative study. How fast do they work inside the cell? How much are they regulated by receptor signaling? We made a study of ER–plasma membrane junctions where the normal Sac1 4-phosphatase enzyme intrinsic to ER membranes can regulate plasma membrane PtdIns(4,5)P_2_ (Dickson et al., 2016). When the ER was chemically recruited next to the plasma membrane, we saw plasma membrane PH_PLCδ1_ decreasing (half time 140 s) through the activity of the ER-endogenous Sac1. If Sac1 was overexpressed, then the depletion upon chemical recruitment of the ER was stronger, and the half time was shorter (∼50 s); in addition, the resting total cellular PtdInsP_2_ measured by mass spectrometry fell by 50%. If either Sac1 or extended synaptotagmin 2 were knocked down with siRNA, the total cellular PtdInsP and PtdInsP_2_ increased. Such observations highlight the importance of interactions across membrane junctions in controlling plasma membrane phosphoinositides.

## Conclusions and outlook

This essay has emphasized kinetic aspects of PtdIns(4,5)P_2_ signaling that are due to receptor activation, local lipid metabolism, and transport of phosphoinositides among cellular compartments. During receptor responses, the polyphosphoinositide pools show wide modulation as do proteins dependent on them. The primary message is that everything can be remarkably fast and there is brisk homeostatic regulation that restores the resting state when the stimulus is gone—and even when the stimulus persists. Breakdown by PLC takes only a few seconds. After agonist is removed, resynthesis from PtdIns takes one or a few minutes depending on the cell type. Synthesis of new pools of phosphoinositides to complete the “PI cycle” all the way from adding *myo*-inositol to diacylglycerol takes <10 min. During continued stimulation of G_q_-coupled receptors, the lipid kinase steps (and perhaps other supporting activities) that synthesize PtdIns(4,5)P_2_ are accelerated in several stages, shifting from a low-gear maintenance mode to a high-gear recovery mode that can speed PtdIns(4,5)P_2_ production by more than 100-fold. These steps and their regulation have been vetted by mathematical modeling. The regulators of the lipid kinases are powerful but not yet sufficiently known. They may include physical recruitment and scaffolding of the lipid kinase pathways and inter-compartmental lipid transfers, as well as more conventional phosphorylation or allosteric control of the individual enzymes.

Our work has focused on the physiology of phosphoinositides in mammalian cells. We have established methods to measure signaling events mediated by the PtdIns(4,5)P_2_ pathway and have learned more about how signaling by phosphoinositides functions in living cells. It is well known that PtdIns(4,5)P_2_ and the other phosphoinositides serve essential housekeeping functions in every cell, so defects in their metabolism are linked to disease including severe errors in development (Burke 2018; McCrea and De Camilli, 2009; Hammond and Burke, 2020; Vivas et al., 2019; Cao et al., 2020). The mechanistic insight we have obtained contributes to our understanding of disease pathophysiology and can guide investigation of novel therapeutic strategies. Many aspects of signaling will be different in the specific cell types affected by the disease, but the approaches and methods we have established will allow further research to test hypotheses and understand signaling in each physiological milieu. A great deal is understood at the molecular and single-cell level. There the field is very mature. Above the single-cell level, we have outlined the importance of PtdIns(4,5)P_2_ to modulate both neuronal “attention” and vascular tone, but we feel that much more remains to be learned. We look forward to continued investigation of the phosphoinositide dynamics underlying physiological and pathophysiological phenomena and the new therapeutic strategies that may result.
